# Effects of Sperm Conjugation and Dissociation on Sperm Viability *In Vitro*

**DOI:** 10.1371/journal.pone.0034190

**Published:** 2012-03-30

**Authors:** Dawn M. Higginson, Kali R. H. Henn

**Affiliations:** Department of Biology, Syracuse University, Syracuse, New York, United States of America; University of Otago, New Zealand

## Abstract

Sperm conjugation is an unusual variation in sperm behavior where two or more spermatozoa physically unite for motility or transport through the female reproductive tract. Conjugation has frequently been interpreted as sperm cooperation, including reproductive altruism, with some sperm advancing their siblings toward the site of fertilization while ostensibly forfeiting their own ability to fertilize through damage incurred during conjugate break-up. Conversely, conjugation has been proposed to protect sensitive regions of spermatozoa from spermicidal conditions within the female reproductive tract. We investigated the possibility of dissociation-induced sperm mortality and tested for a protective function of conjugation using the paired sperm of the diving beetle, *Graphoderus liberus*. Sperm conjugates were mechanically dissociated and exposed to potentially damaging tissue extracts of the female reproductive tract and somatic tissue. We found no significant difference in viability between paired sperm and dissociated, single sperm. The results further indicate that the reproductive tract of female *G. liberus* might not be spermicidal and conjugation is not protective of sperm viability when damaging conditions do exist. Our results support the interpretation that, at least in some taxa, sperm conjugation is neither protective nor damaging to sperm viability.

## Introduction

Sperm conjugation is a rare, but taxonomically widespread, adaptation to postcopulatory sexual selection where two or more spermatozoa physically unite for motility or transport through the female reproductive tract before dissociating prior to fertilization [1]. Conjugation has frequently been interpreted as cooperation, with some sperm purportedly acting as reproductive altruists, foregoing fertilization opportunities to enhance the probability of fertilization by their sibling sperm [2], [3], [4], [5], [6] (but see [7]). Conjugates are stabilized by cell-cell or cell-matrix interactions, and while the mechanisms of conjugate dissociation are unknown, the break-up of such intimately associated cells might disrupt cell membrane integrity resulting in a loss of fertilizing ability or death of a proportion of the participating sperm while leaving others unharmed [7].

In several species of rodents, sperm heads have reflexed apical hooks that open after ejaculation and become entangled with other sperm to form large, disorganized conjugates known as sperm trains [5], [8], [9]. While dissociation has not been investigated in most species, in the wood mouse, *Apodemus sylvaticus*, conjugate break-up is concomitant with premature acrosome reaction by approximately half of the participating sperm [5]. Similarly, the break-up of the paired sperm of the opossum, *Didelphis virginiana*, *in vitro* is associated with loss of motility by one of the participants, a critical indicator of a sperm's ability to fertilize an egg [6] (but see [10]). The damage observed in the wood mouse and the opossum has been interpreted as evidence of a fitness cost to sperm caused by conjugate dissociation [5], [6]. In species where cell surface interactions are important for the formation or stabilization of conjugates, it seems probable that death of participating sperm would lead to conjugate break-up [7]. Likewise, where sperm conjugates are hydrodynamically synchronized (the expected condition), theoretical models suggest that development of disparity in sperm beat frequencies, such as would be the case with weak or dying sperm, would result in conjugate break-up [11]. There is no a priori expectation, however, that sperm mortality is a necessary consequence of conjugate dissociation and sperm damage caused by unrelated reasons (e.g., spermicidal environments or age) might explain the observed co-occurrence of a loss of viability and conjugate break-up. An experimental approach is necessary to distinguish between the alternate scenarios of sperm mortality contributing to conjugate break-up or sperm death as a required mechanism for conjugate dissociation.

Conversely, conjugation has also been proposed to protect sensitive regions of sperm (e.g., the acrosome) from damaging conditions within the female [12], [13]. For example, the sperm of guinea pigs form orderly stacks that separates the acrosomes of all but the top most spermatozoa from the external environment [14], [15] and may thereby protect them from degradation. Likewise, in *D. virginiana* sperm are tightly apposed along the acrosomal surface, effectively forming a protective seal around the potentially fragile acrosomes [12]. In many species, however, sperm conjugation leaves the acrosome exposed (e.g., some diving beetles [16], [17]; rodents [9], [18]). Moreover, conjugation has the potential to be protective of sperm viability only to the extent that conditions within the female reproductive tract are damaging [19], [20].

Here we investigate the possibility of dissociation-induced sperm mortality and test for a protective function of conjugation against spermicidal conditions in the diving beetle *Graphoderus liberus*. The sperm of *Graphoderus* pair with other spermatozoa lying in close proximity within the seminal vesicles of males [21]. Sperm remain conjugated during travel through the female reproductive tract and in storage, only dissociating when near to the site of fertilization [22]. The sperm protection hypothesis for the function of conjugation would be supported if, i) the female reproductive tract is spermicidal and ii) conjugation reduces sperm mortality in spermicidal conditions. Likewise, if ‘normal’ conjugate dissociation is achieved through a mechanism that damages one or more of the participating sperm, in species with paired sperm such as *G. liberus*, this would result in 50% of all single sperm being inviable. We mechanically dissociated sperm pairs and used diagnostic fluorescent staining to distinguish between live sperm cells with intact cell membranes and sperm with damaged cell membranes that result in sperm death. The results indicate that conjugation was neither protective in damaging environments, nor was conjugate break-up associated with increased sperm mortality.

## Results

**Figure 1:**
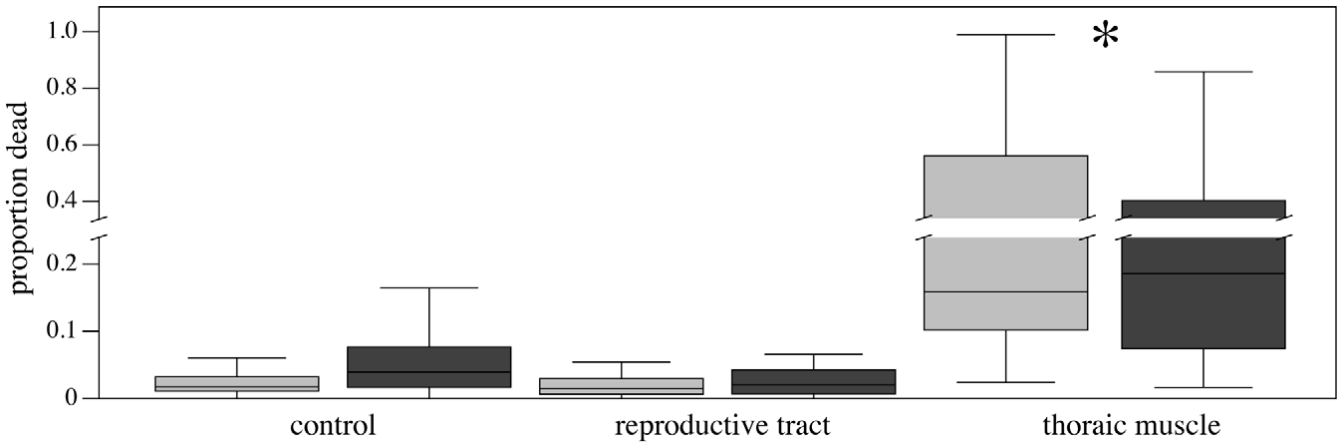
Proportion of dead sperm after one hour of exposure to Grace's medium, female reproductive tract extract in Grace's medium (control) or thoracic muscle extract in Grace's medium. Sperm exposed to thoracic muscle extract had significantly higher mortality (indicated by *), regardless of whether sperm were conjugated or single, than the other treatments (orthogonal contrast: t_229_ = 5.69, p<0.0001). Box plots show median, 25% and 75% quartiles; whiskers indicate the interquartile range. Light grey = single sperm, dark grey = conjugated sperm.

Within the seminal vesicles of males (n = 23), most sperm were paired (median proportion: 0.94), although the proportion of conjugated sperm varied dramatically among males (range: 0–0.99), and had low rates of mortality (median proportion: 0.01, range of 0–0.06). A subsample of each male's sperm was vortexted to mechanically disrupt sperm pairs. Using a fully-factorial experimental design, single and conjugated sperm from each male were exposed to three treatment solutions, i) insect tissue culture medium, ii) female reproductive tract extract, and iii) thoracic muscle extract to control for exposure to foreign tissue (see Materials and Methods). Mechanically-induced conjugate break-up was not significantly associated with sperm mortality (F_1,229_ = 0.29, p = 0.59), nor was there an interaction between conjugation status and treatment (F_2,229_ = 0.16, p = 0.85). However, treatment solution significantly influenced sperm mortality (F_2,229_ = 13.90, p<0.0001; Fig. 1). Female reproductive tract extract was not spermicidal (i.e., no difference in mortality between sperm exposed to Grace's medium and reproductive tract extract, t_229_ = 1.01, p = 0.31). Unexpectedly, thoracic muscle extract increased sperm mortality when compared to the other treatments (t_229_ = 5.69, p<0.0001). Sperm conjugation was not protective when sperm were exposed to spermicidal environments (i.e., thoracic muscle extract; F_2,229_ = 0.16, p = 0.85; Fig. 1).

## Discussion

Here, we tested two non-mutually exclusive hypotheses regarding sperm conjugation, i) conjugation is protective of sperm viability in damaging environments, and ii) conjugation reduces viability of some participating sperm due to the mechanism of conjugate break-up. The results failed to support either hypothesis. To the extent that experimental conditions reflect the naturally occurring chemical environment, the female tract of *G. liberus* is not spermicidal. Moreover, conjugated sperm exposed to damaging conditions died in the same proportion as single sperm and at a rate far lower than the 50% mortality predicted if the death of one sperm was required for pair break-up. Additionally, mechanical dissociation of sperm conjugates resulted in single sperm populations with low levels of mortality comparable to that of unmanipulated sperm samples consisting primarily of paired sperm.

Spermicidal reproductive tracts are common in birds, mammals and invertebrates, and might serve to protect females from infection, prevent polyspermy, provide nutrients from digested sperm or permit females to bias fertilization in favor of robust or preferred sperm [19], [20]. Tissue extracts are an imperfect representation of the chemical environment that sperm typically experience within females, however, similar tissue extract methodology has positively identified spermicidal conditions in *Drosophila pseudoobscura* where the results were confirmed by *in vivo* observations [23]. Maintenance of sperm viability during prolonged periods of storage is mediated through sperm-female interactions, as females must provide protection and nutrition to the sperm they harbor [24]. Thus, based on results presented here and opportunistic observations of viable sperm harvested from the spermatheca of field collected females that were ‘overwintered’ at 4°C for 5 months (n = 11, median proportion viable 0.48, range 0.11 to 0.63), we tentatively conclude that the reproductive tracts of *G. liberus* are not spermicidal.

Membrane disruption caused by conjugate break-up has been proposed as a fitness cost associated with sperm conjugation [5], [6], however, the mechanism of conjugate dissociation has not been investigated for any species [7]. In most diving beetles, sperm remain conjugated until positioned for fertilization [22] (and personal observations). Conversely, the paired sperm of *Dytiscus marginalis* have been observed to dissociate within the spermatheca [21]. Although a glycocalyx covering a portion of the sperm tail was lost, and the plasma membrane adjacent to the nucleus was ‘wavy’, transmission electron microscopy reveal no perceptible signs of sperm damage associated with conjugate dissociation [21]. It is probable that the mechanical dissociation of conjugates reported here would be more damaging than natural mechanisms, yet we observed no reduction in sperm viability in *G. liberus* associated with conjugate break-up. While there have been intriguing suggestions that conjugation might represent cooperation among sibling sperm in rodents [8] (but see [25], [26]), to date there has been no convincing demonstration of fitness costs of conjugation to individual sperm in any species and accordingly, no evidence of altruistic behavior among sperm [3], [7], [27].

## Materials and Methods

### Beetles

*Graphoderus liberus* were field collected, separated by sex and transported to the laboratory where they were held in aquaria and fed freeze-dried mealworms and crickets *ad libitum*. The beetles were reproductively active, with almost all females having stored sperm. To obtain tissue and sperm samples, beetles were euthanized with ether and dissected in supplemented 1× Grace's insect tissue culture medium (Invitrogen).

### Ethics Statement

All necessary permits were obtained for the described field studies (New York State Fish and Wildlife License to collect or possess 321). Beetles were collected at the Cornell Research Ponds by permission of the manager, Robert Johnson.

### Sperm mortality assay

**Figure 2:**
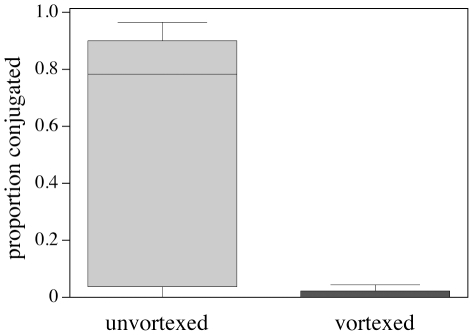
Proportion of paired sperm after one hour in Grace's medium for vortexed and unvortexed sperm samples. Vortexing effectively dissociated sperm conjugates (Wilcoxon signed rank test, p<0.0001; n = 47, median proportion conjugated: 0.02, range 0–0.88 versus unvortexed median: 0.78, range 0–0.97). Box plots with interquartile whiskers.

Sperm from the seminal vesicles of each male (n = 47) were exposed to every treatment. Single sperm were obtained by vortexing a subsample of a male's sperm at maximum speed for 30 seconds (Fig. 2). Tissue extracts were prepared by dissecting and pooling the relevant tissue (n = 52 females, i.e., one tract per male plus extra to account for solution loss), flash freezing, and grinding to release cell contents. Specifically, the female tissue designated as ‘reproductive tract’ included the spermatheca and fertilization duct but excluded the bursa as males can potentially deposit sperm directly into the storage organ (see [22], [28] for a description of female reproductive morphology; terminology used as per [22]). Tissues were pooled negate potential differences due to variation in females (e.g., age or time since previous mating). Subsequently, the equivalent of 6.5 µl Grace's medium per female was added to the ground tissue, mixed thoroughly, and briefly centrifuged at 11,100 rcf to pellet cellular debris. The supernatants were stored at −40°C until use.

Single and conjugated sperm were divided into 6 µl aliquots and incubated at room temperature with an equal amount of treatment solution. After one hour, 2 µl of LIVE/DEAD stain was added (40 µl Grace's medium, 2 µl 2.4 mM propidium iodide (red = dead), 1 µl of 1 mM SYBR® 14 dye (green = live), Molecular Probes LIVE/DEAD® sperm viability kit) and 10 µl of sperm suspension plus stain was transferred to a microscope slide for imaging. Three locations per slide were chosen haphazardly, and imaged at 200× with DIC microscopy and epifluorescence using a Semrock GFP/DSRED dual pass filter. An observer, blind to the treatment groups, counted live/dead and single/conjugated sperm (per treatment mean of 217 sperm, 95% CI 207 to 227). The experiment was replicated twice (*n* = 24 and 23 respectively). In replicate two, we additionally examined unvortexed sperm immediately after harvest to determine proportion of viable and conjugated sperm. Experiments were completed within four days of beetle collection.

### Statistical analyses

Analyses were performed in SAS 9.1.3 and JMP 9. Proportion dead sperm per treatment were arcsine square-root transformed and a mixed model ANOVA was used to test for differences among treatments. Because there were repeated measures from each male, ‘individual’ was added as a random effect using a compound symmetrical covariance-structure. There were no significant differences in sperm mortality between the experimental replicates with respect to treatment (p>0.23) and they were thus combined for subsequent analyses. Orthogonal contrasts were used to test for main and simple treatment effects. Randomization tests confirmed our findings, indicating that violation of ANOVA's normality of assumption did not impact the results.
